# Provider kinematic strategies during the delivery of spinal manipulation and mobilization: a scoping review of the literature

**DOI:** 10.1186/s12998-024-00564-x

**Published:** 2025-01-06

**Authors:** Katie Svoboda, Samuel J. Howarth, Martha Funabashi, Lindsay M. Gorrell

**Affiliations:** 1 Department of Graduate Education, Canadian Memorial Chiropractic College, Toronto, ON Canada; 2 Division of Research and Innovation, Canadian Memorial Chiropractic College, Toronto, ON Canada; 3 Department of Chiropractic, Université du Québec à Trois-Rivières, Trois-Rivières, Canada; 4 Research Center, Parker University, Dallas, TX USA; 5 Department of Chiropractic Medicine, Integrative Spinal Research Group, Balgrist University Hospital, University of Zurich, Zurich, Switzerland

**Keywords:** Spinal manipulation, Spinal mobilization, Biomechanics, Kinematics, Spine pain

## Abstract

**Background:**

Spinal manipulation (MAN) and mobilization (MOB) are biomechanically different yet both elicit pain reduction and increased range of motion. Previous investigations have focused on quantifying kinetics (e.g., applied forces) or, recipient kinematics (i.e., movements) during MAN and MOB. While these studies provide valuable information, they do not report on the strategies adopted by providers when performing the complex motor tasks of MAN and MOB. This review sought to synthesise the literature reporting on provider kinematics during the delivery of MAN and MOB.

**Methods:**

This scoping literature review is reported following the Preferred Reporting Items for Scoping Reviews (PRISMA-ScR) statement. MEDLINE (Ovid), PsychINFO, Cochrane Library, Web of Science, Embase, Scopus, PEDro, ICL and CINAHL databases were searched from inception to September 2023 for terms relating to provider kinematics during the delivery of MAN and MOB. Data were extracted and reported descriptively, including: general study characteristics, number and characteristics of individuals who delivered/received MAN and/or MOB, region treated, equipment used and kinematic parameters of the individual delivering the procedure.

**Results:**

Of 4,844 records identified, five (0.1%) fulfilled the eligibility criteria and were included in the analysis. Of these, provider kinematics were reported for the delivery of MAN in four (80%) and for the delivery of MOB in one (20%) article. Practitioners applied the procedure in all (100%) and students in one (20%) study. Spinal regions treated were: lumbar (*n* = 4), thoracic (*n* = 2) and cervical (*n* = 1). Data were reported heterogeneously but were most commonly captured using either video or motion capture equipment (*n* = 4, 80%). The direction of applied force was fully reported in one (20%) and only partially reported (one spinal region) in another study.

**Conclusions:**

There are a small number of studies reporting heterogeneously on provider kinematics during the delivery of MAN and MOB. Clear reporting of the procedure from a biomechanical perspective and of the measurement equipment used could enable future meta-analysis of provider kinematic data, the use of provider kinematic data in the development of technique skills curricula and could feasibly be used to mitigate risk of injury for providers.

**Supplementary Information:**

The online version contains supplementary material available at 10.1186/s12998-024-00564-x.

## Introduction

Conservative interventions such as spinal manipulation (MAN) and spinal mobilization (MOB) are recommended and effective manual therapies that are commonly used in treatment plans for managing spinal disorders [1–4]. Performing either MAN or MOB is a complex psychomotor skill that requires whole-body coordination as the provider applies time-varying forces, with different characteristics, to the recipient [5]. Previous investigations have predominantly focused on quantifying force-time characteristics of a provider’s performance (e.g., magnitude of applied forces) of MAN [6] and MOB [7]. While these studies provide valuable information about the delivery of MAN and MOB, they do not report on the kinematic strategies adopted by providers when applying these procedures [8, 9]. A quantitative understanding of kinematic strategies used by those applying either MAN or MOB can inform the development and teaching of manual therapy skills curricula and may offer relevant information to mitigate injury risk [10–12].

Contemporary teaching of MAN and MOB skills includes a combination of theory-based lectures, instructor-guided mimicry and practice with performance feedback devices [9]. Theory-based lectures and instructor-guided mimicry for teaching MAN and MOB often use textbook images of clinician posture and hand contacts, coupled with narrative descriptions for the intended movements of both the recipient and provider [13]. Relatedly, two Delphi studies attempted to outline critical competencies for applying manual therapy and to recommend postures of both the recipient and provider for MAN and MOB technique educators to focus on [14, 15]. Participants in these studies were either manual therapy educators and/or members of the American Association for Orthopedic Manual Physical Therapists. Identified attributes addressed biomechanical aspects of the provider’s technique such as posture/movement (e.g., body position over the top of the recipient during the preparatory phase, dropping downwards during the impulse phase) and force generation (e.g., use of forearms to maintain contact/generate force during the preparatory phase, generation of force through the body and legs during the impulse phase). Effectively, these studies and the current approach to teaching MAN and MOB skills contributes to the transfer of baseline information for the body postures and movements of the provider from one generation of practitioners to the next [16]. However, they do not provide a quantitative description of the actual postures and movements of the provider’s body as they perform manual therapy.

Moreover, several cross-sectional studies have reported that MAN and MOB delivery is an occupational activity of manual therapy providers that can contribute to the development of musculoskeletal issues in the hand/wrist, shoulder and lower back [10–12, 17]. Specifically, participants attributed their manual therapy-related musculoskeletal issues to having to impart forces to a recipient’s body while adopting awkward postures. To this point, previous work demonstrated that increasing table height during MAN delivery can significantly reduce spine flexion and low-back compression, two biomechanical variables that are often associated with an increased risk of low back disorders [18]. The potential for injuries related to provider biomechanics of MOB delivery has also been reported [19–21]. For example, force application with an awkward thumb position in students could be a catalyst for thumb injury [19]. This hypothesis is further supported by the data reporting that 88% of physiotherapists modify their manual therapy techniques due to pain and 22.7% suffer from thumb osteoarthritis and radial-side wrist joint dysfunction [20]. In an attempt to reduce the risk of injury, taping of the thumbs prior to MOB was reported to improve thumb alignment in a cohort of physiotherapy students [21]. Understanding the kinematic strategies used by providers performing MAN and MOB may inform the development of approaches that minimise the biomechanical features that could contribute to musculoskeletal issues.

Collectively, these studies highlight the importance of measuring and quantifying provider kinematics during the application of both MAN and MOB. As such, the objective of this scoping review was to synthesise the literature reporting on provider kinematics during the delivery of MAN and MOB.

## Main text

### Methods

This scoping literature review was conducted in 5 stages as outlined by Arksey and O’Malley [22]. Specifically: (i) identification of the research question; (ii) identification of potentially relevant studies; (iii) selection of relevant studies; (iv) charting of data; and (v) generating results by collating, summarizing and reporting the data. The final optional consultation process step was not included as it was deemed to be unnecessary in the context of the current study. The Preferred Reporting Items for Scoping Reviews (PRISMA-ScR) statement was used to report the data [23]. The protocol was designed by an international and interprofessional team of chiropractors, physiotherapists and scientists with relevant methodological, biomechanical and clinical expertise and was prospectively registered with the Open Science Framework Registry (https://osf.io/4vtgx/). Ethical approval was not required.

### Eligibility criteria

Criteria for studies, retrieved using our search strategies (described in the next section), to be included and excluded in this review are listed in Table 1.

**Table 1:** Eligibility criteria

Category	Inclusion criteria	Exclusion criteria
Language	English	All other languages
Individual applying the procedure	Adults (*≥* 18 years)	Individuals outside of this age range (< 18 years)
Procedure	MAN and/or MOB	Other manipulation/mobilization techniques (e.g., instrumented manipulations, assisted MAN and/or MOB, surgical joint manipulation, post-surgical passive mobilization, etc.)
Outcomes	Kinematic variables (e.g., joint angles, joint velocity, change in centre of mass, etc.)	Kinetic variables (e.g. peak force, ground reaction force, rate of force production, etc.)
Study design	Randomized controlled trials, cohort studies, case control studies, case series/reports, observational cross-sectional studies	Editorials, conference proceedings, commentaries, letters to the editor, expert opinion articles, secondary sources (e.g., textbooks, etc.)

Abbreviations: MAN: spinal manipulation; MOB: spinal mobilization

### Search strategy

The following databases were searched from inception to September 18th 2023: MEDLINE, PsychINFO, Cochrane Library, Web of Science, EMBASE, Scopus, PEDro, Index to Chiropractic Literature and CINAHL. The search strategy was developed by the authors with the assistance of two experienced health sciences librarians. The initial search strategy was developed with Ovid MEDLINE using medical subject headings (MeSH): Manipulation, Chiropractic/, Manipulation, Spinal/, Manipulation, Orthopedic/, Musculoskeletal Manipulations/, Manipulation, Osteopathic/, Biomechanical Phenomena/, Physical Phenomena/, Motor Skills/; and text words: chiropractor, osteopath, naprapath, physiotherapist, spine, manual, lumbar, cervical, thoracic, pelvic, high-velocity low-amplitude, low-velocity low-amplitude, kinematic. This search strategy was subsequently adapted to the syntax and subject headings of the other databases that were searched. Search strategies for all databases are provided in Appendix 1.

### Study selection process

Records retrieved from the electronic searches were de-duplicated in Zotero (v6.0.30) prior to export to the Rayyan platform (2022) [24]. As a first screening step, two authors (KS and LG) independently screened the titles and abstracts of the identified records against the inclusion and exclusion criteria. Following this, the same two authors then screened the full texts of potentially relevant records identified during the title and abstract screening step. Any disagreements were resolved by discussion and consensus between the two reviewers. A third author (SH) was consulted if consensus could not be reached.

### Data extraction

Data from eligible articles were independently extracted by two authors (KS and LG). Extracted data included: study characteristics (e.g., year of publication, design) and characteristics of the individual delivering the procedure (e.g., profession, experience), location of data collection (e.g., country, institution), number of individuals delivering MAN and/or MOB, the joint to which the procedure was applied, equipment used for measurements (e.g., motion capture system), region treated (e.g., thoracic/lumbar spine) and kinematic variables (e.g., joint angles, joint velocities, centre of mass position).

### Data synthesis

Proportions and frequencies of studies reporting on each of the previously specified domains were calculated as a descriptive synthesis of the data (Excel, Microsoft Corp., Redmond, WA, USA). Study quality was not assessed due to considerable heterogeneity of the reported data and the descriptive nature of this scoping review.

## Results

**Fig. 1:**
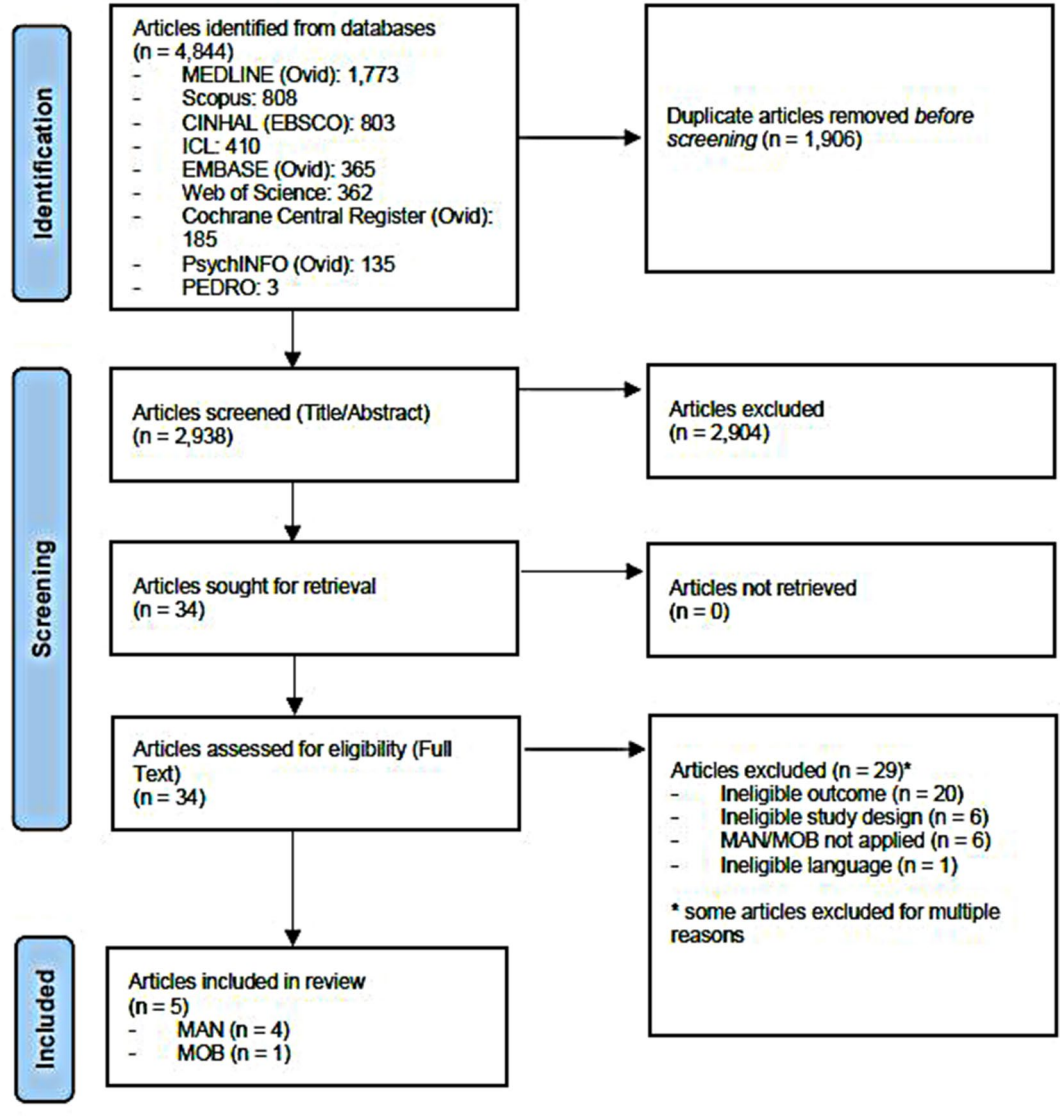
PRISMA flow-chart

The electronic searches identified 4,844 records, with 2,938 records remaining after de-duplication (*n* = 1,906) (Fig. 1). Titles/abstracts of these 2,938 records were screened, which yielded 34 articles for full-text screening. Twenty-nine full-text articles were excluded (e.g., MAN/MOB not applied, kinematic data of provider not reported) and are listed in Appendix 2, leaving 5 included articles for data extraction.

### Characteristics of included studies

The five included articles were published between 2002 and 2022 and all used an observational cross-sectional design (Table 2). Data were collected in North America (n = 4) and Australia (n = 1). Four (80%) studies reported on MAN [18, 25–27] and one (20%) reported on MOB [28]. MAN was applied to the lumbar (n = 3), thoracic (n = 2) and cervical (n = 1) spine of humans. In the one study reporting on MOB, the procedure was applied to the lumbar spine of humans. The procedure was most commonly applied by health care providers (n = 5) to human adults (n = 4) at academic institutions (n = 5). The applied ‘technique’ was named in all but one study ([18], technique reported only for the thoracic spine), with the position of the MAN/MOB recipient reported in four studies (80%) and the direction of applied force being reported in only one (20%) study ( [18], only reported for the thoracic spine).

**Table 2:** Characteristics of included studies

Author/s year, country	Study design	MAN/ MOB	Individual delivering procedure Experience, *n*	Individual receiving procedure *n*	Region treated	Applied procedure description		
						Force direction	Recipient position	Procedure named
Bereznick et al. 2002, Canada (25)	Observational cross-sectional	MAN	Chiropractors > 5 year, *n* = 9	Unclear *n* = 5	Thoracic	Yes	Yes	Yes
Lorme & Naqvi 2003, Australia (18)	Observational cross-sectional	MAN	Chiropractors Mixed, *n* = 7	Adult *n* = 1	Cervical Thoracic Lumbar	Cervical: no Thoracic: yes Lumbar: no	No	Cervical: no Thoracic: yes Lumbar: no
Derian et al. 2020, USA (26)	Observational cross-sectional	MAN	Physiotherapists Mixed, *n* = 4 Students NR, *n* = 39	Adult NR	Lumbar	No	Yes	Yes
Mehyar et al. 2020, USA (28)	Observational cross-sectional	MOB	Physiotherapists Mixed, *n* = 2	Adult *n* = 16	Lumbar	No	Yes	Yes
Weiner et al. 2022, USA (27)	Observational cross-sectional	MAN	Chiropractor > 5 year, *n* = 1	Adult *n* = 10	Lumbar	No	Yes	Yes

Abbreviations: Adult: adult human (18–65 years old); MAN: spinal manipulation; Mixed: practitioner experience both < and > 5 years; MOB: spinal mobilization; n: number; NA: not applicable; No: information not reported; NR: not reported; Yes: information reported; yr: years

### Spinal manipulation

Extracted information from the four studies that reported provider kinematics while performing MAN is provided in Table 3 [18, 25–27]. Provider kinematics were quantified using video (n = 2), motion capture and a ‘Lumbar Motion Monitor’ (n = 1) and inertial motion units (n = 1). In the earliest study reporting on provider kinematics during MAN, Bereznick and colleagues (2002) measured average linear hand displacements (inferior to superior) during thoracic MAN of (mean ± SD) 38.75 ± 12.3 mm and 33.25 ± 8.5 mm when ‘hooking’ the transverse and spinous processes, respectively [25]. Investigating cervical, thoracic and lumbar MAN, Lorme and Naqvi (2003) reported that maximum sagittal flexion of the lumbar spine differed as workstation table height was changed [18]. Furthermore, there were differences in lumbar spine maximum sagittal flexion and axial rotation velocity during cervical, thoracic and lumbar MAN. In the lumbar spine, Derian and colleagues (2020) reported that experts (compared to novices) exhibited greater peak pelvic angular velocity in the frontal plane, with all experts tilting the right side of their pelvis inferiorly. In contrast to this, novices tended to tilt the right side of their pelvis superiorly. Similarly, in the transverse plane, experts had a greater peak pelvic angular velocity and displayed cephalic pelvic rotation (towards the recipient’s head) compared to novices who tended to rotate their pelvis caudally. There were no differences in peak angular velocity in the sagittal plane [26]. Furthermore, experts had greater downward pelvic linear velocity compared to third- and first-year students. Also investigating provider kinematics during MAN delivered to the lumbar spine, Weiner and colleagues (2022) reported that a single provider typically displayed a movement pattern of flexion and left lateral flexion in all spinal regions, with variable left/right axial rotation at the onset of MAN delivered to 10 recipients [27]. Provider movement patterns were reported by the authors to be ‘characterized by biphasic wavelike motions’, with similarities among recipients during the thrust but inconsistent thrust timings. Similarly, angular velocities were variable throughout the spine but were largest in the cervical and thoracic regions.

**Table 3:** Description of spinal manipulation studies

Author/s year, country	Kinematic variable assessed	Measurement equipment Metrological details reported?	Main results
Bereznick et al. 2002, Canada (25)	Linear displacement of the hands	Video tape No	Linear (inferior to superior) displacement of provider hands: - Transverse process hook: mean (SD), range – 38.75 (12.3), 12.5 to 70.0 mm - Spinous process hook: mean (SD), range – 33.25 (8.5), 15.0 to 45.5 mm
Lorme & Naqvi 2003, Australia (18)	Maximum sagittal flexion, axial rotational velocity, and maximum lateral velocity of lumbar spine	Video camera & ‘Lumbar Motion Monitor’ No	Workstation table height: - Significant difference for MSF as workstation table height was changed (F: 26.462, *p* = 0.002) - No significant difference for ARV (F: 0.007, *p* = 0.993) or MLV (F: 0.021, *p* = 0.979) - Significant difference between low and medium (F: 16.4, *p* = 0.007), medium and high (F: 27.0, *p* = 0.002), and low and high (F: 63.3, *p* = 0.000) for MSF - Lumbar MAN: medium vs. low table height reduced MSF by 13.9% (2.66 N^*^) - Thoracic MAN: medium vs. low table height reduced MSF by 35% (10.4°) - Cervical MAN: medium vs. low table height reduced MSF by 6% (2.2°); medium vs. high table height reduced by 22.7% (8.1°); low vs. high table height reduced MSF by 27.3% (10.3°) MAN tasks: - Significant difference for MSF (F: 52.701, *p* = 0.000), ARV (F: 26.993, *p* = 0.002), dominant elbow moment (F: 27.688, *p* = 0.002), and dominant shoulder moment (F: 20.165, *p* = 0.004) - Significant difference for MSF between lumbar and thoracic MAN (F: 93.4, *p* = 0.000) and thoracic and cervical MAN (F: 36.4, *p* = 0.001) o MSF was 32.8% (9°) less for thoracic vs. lumbar MAN o MSF was 43.8% (14.8°) less for thoracic vs. cervical MAN - Significant differences between lumbar and thoracic MAN (F: 33.8, *p* = 0.001) and thoracic and cervical MAN (F: 11.4, *p* = 0.015) and lumbar and cervical MAN (F: 60.5, *p* = 0.000) for ARV o Cervical (2.086°/s) vs. lumbar (4.295°/s) MAN reduced ARV by 51% (regardless of table height) o Cervical (2.086°/s) vs. thoracic (2.576°/s) MAN reduced ARV by 19% o Thoracic (2.576°/s) vs. lumbar (4.295°/s) MAN reduced ARV by 40% - MLV for lumbar MAN higher (28.6°/s) than for thoracic (21.4°/s) or cervical (21.5°/s) MAN
Derian et al. 2020, USA (26)	Peak angular and linear velocity of the pelvis	MoCap No	- Experts exhibited higher peak pelvic angular velocity compared to novices in the frontal plane (*p* = 0.020) and transverse plane (*p* = 0.000) - Experts demonstrated greater downward pelvic linear velocity than third-year students and first-year students (*p* = 0.000)
Weiner et al. 2022, USA (27)	Angular deviation and angular velocities of cervical, thoracic and lumbar spine	IMU No	Cervical: movement patterns (angular deviation)^#^; angular velocities - Sagittal: flexion, extension, flexion, extension (range: 26.0 to 2.9°); range: 137.3 to -170.3°/s - Frontal: left lateral flexion, right lateral flexion, left lateral flexion (range: 10.2 to -2.1°); 134.3 to -47.7°/s - Transverse: right axial rotation, left rotation after thrust (range: -6.0 to -16.9°); 205.8 to -75.1°/s Thoracic: movement patterns during MAN; angular velocities - Sagittal: flexion, extension, flexion, extension, flexion (range: 24.6 to 4.1°); range: 99.6 to -165.6°/s - Frontal: left lateral flexion, right lateral flexion, left lateral flexion (range: 6.0 to -12.2°); range: 140.3 to -127.7°/s - Transverse: right axial rotation, left or right axial rotation (range: 13.5 to -10.0°); range: 113.0 to -155.7°/s Lumbar: movement patterns during MAN; angular velocities - Sagittal: flexion, extension, oscillation flexion/extension (range: 41.9 to -23.7°); range: 48.7 to -69.2°/s - Frontal: left or right lateral flexion, left lateral flexion, right lateral flexion (range: 7.6 to -10.5°); range: 63.7 to -24.8°/s - Transverse: left axial rotation, (mostly) right axial rotation, left axial rotation (range; 20.3 to -2.8°); range: 49.5 to -100.0°/s

Abbreviations: ARV: axial rotation velocity; IMU: inertial measurement unit; MAN: manipulation; MLV: maximum lateral velocity; MSF: maximum sagittal flexion; MoCap: optoelectronic motion capture; p: p-value; s: seconds; SD: standard deviation; *: as reported in the original manuscript; #: absolute range reported from setup to resolution after thrust

### Spinal mobilization

Extracted information from the one study that reported provider kinematics while applying MOB is provided in Table 4 [28]. Provider hand displacements (posterior to anterior) were quantified using motion capture and inertial measurement units at four different levels of applied force during the delivery of lumbar spine MOB. Hand displacement amplitude increased with increasing force application: (mean ± SD) 30 N – 1.7 ± 0.2 mm; 60 N – 3.3 ± 0.5 mm; 90 N – 5.5 ± 0.9 mm; and 120 N – 7.7 ± 1.2 mm.

**Table 4:** Description of spinal mobilization study

Author/s year, country	Kinematic variable assessed	Measurement equipment Metrological details reported?	Main results
Mehyar et al. 2020, USA (28)	Hand displacement	IMU & MoCap Yes	The mean amplitude ± SD of displacements were: 30 N: 1.7 ± 0.2; 60 N: 3.3 ± 0.5; 90 N: 5.5 ± 0.9; 120 N: 7.7 ± 1.2 mm. The mean difference in the amplitude of displacement between the IMU and the MoCap system was less than 0.3 mm.

Abbreviations: IMU: inertial measurement unit; MoCap: optoelectronic motion capture; N: Newton; SD: standard deviation

## Discussion

This review synthesised the literature reporting on provider kinematics during the delivery of MAN and MOB and highlights a paucity of investigation in this area, coupled with considerable variability in measurement and reporting of the outcomes of interest. The few studies that do report in this area have provided a description of provider spine kinematics during MAN [18, 26, 27] and displacement of the providers’ hands during MAN [25] and MOB [28]. More specifically, there is large heterogeneity in provider spinal kinematics during MAN which may be influenced by table height [18] and experience level [26]. However, it is likely that many more factors beyond those reported in the current literature (e.g., recipient body morphology, injury status) could also influence provider kinematics. Hand displacements during MAN and MOB are similarly heterogeneous, with larger displacements being measured with greater applied forces during MOB [28]. Collectively, these preliminary findings suggest that provider kinematics during MAN and MOB are influenced by provider experience, the applied procedure and the kinetic input of the provider (e.g., applied force).

Intuitively, provider kinematics during the delivery of MAN and MOB would be influenced by both provider and recipient body morphology. However, such data are somewhat missing from the existing literature. While two studies mentioned either recipient body morphology [25] or reported height and weight of the recipients [27], there was essentially no analysis or discussion of provider kinematics with respect to this element of the provider-recipient interaction. This is salient as provider competencies considered to be part of the proficient delivery of MAN and MOB include: recipient management and control of self and recipient movement and discriminate touch [14] and provider posture/movement (e.g., body position over the top of the recipient during the preparatory phase, dropping downwards to produce force during the impulse phase) and force generation (e.g., use of forearms to maintain contact/generate force during the preparatory phase, generation of force through the body and legs during the impulse phase) [15]. Clearly these factors are related to both provider and recipient morphology and without description of either, it is extremely difficult, if not impossible, for the reader to understand how the reported results might be relevant to them and/or other published data. Furthermore, a recent study reported that chiropractors who were presented with simulated human silhouettes with differing body morphologies (i.e., sex, height and body mass index) adapted their applied forces to ‘match’ with the envisaged recipient body morphology during the delivery of MAN [29]. This suggests that providers modulate MAN forces based on recipient morphology and as such, it is reasonable to believe that provider kinematics might be similarly influenced by recipient morphology. This hypothesis is supported by increasing hand displacements during posterior to anterior lumbar MOB with increasing force application [28]. Specifically, different levels of force would conceivably be applied to recipients with different body morphologies.

Similarly, without description of the morphology of either provider and/or recipient, it is unknown how the reported data might be used to inform the development and teaching of manual therapy skills curricula, or how these data could offer providers relevant information to mitigate the risk of injury during the delivery of both MAN and MOB. As such, detailed description of provider and recipient body morphology is essential for the interpretation and use of data reporting on provider kinematics during the delivery of MAN and MOB. Furthermore, preliminary data suggests that experienced physiotherapists exhibited distinct thumb joint angles compared to novices, suggesting that there could be variation in how experienced providers apply the procedure during their clinical practice [30]. It is possible that these variations are a function of not only level of experience, but also current injury status. However, as MOB was applied to an instrumented tool (6-axis load cell), the reported kinematics may not be entirely consistent with how it is delivered to a human recipient.

Other authors have used ground reaction forces (GRF) to operationalize global (whole-body) coordination during MAN, defining a ‘global coordination index’ as the temporal lag between the onset of force plate unloading (first negative rate of GRF) and the onset of peak force production. Although this ‘global coordination index’ uses kinetic measurements, it has also been used as a performance indicator related to thoracic [31] and lumbar [32] MAN, with data suggesting that experienced providers exhibit greater coordination compared to their novice counterparts. However, it is unknown if and/or how this information has been integrated into the technique skills curricula teaching students MAN. Furthermore, while these studies provide valuable insight into the force-transfer strategies employed by providers during the intervention, they do not provide information regarding the limb and/or torso postures and movement (i.e., provider kinematics). Furthermore, other kinematic approaches (e.g., vector coding, continuous relative phase) could be used to operationalize coordination at the local (joint) level which may be more relevant to the teaching and learning of MAN and MOB.

Regarding injury risk, it has been reported that as treatment table height increases peak lumbar sagittal flexion and disc compressive force decrease [18]. Furthermore, when biomechanical and ergonomic analyses were performed, it was reported that low back compression forces of different transfer tasks (e.g., helping an individual from sitting/side-lying to standing) were greater than a safety threshold but MOB had low to medium risk when considering two custom analyses (Rapid Upper Limb Assessment and Rapid Entire Body Assessment) [33]. At face value, these results suggest that provider body posture is relevant to mitigate injury risk during the delivery of MAN and MOB. However, in the absence of information regarding provider body morphology, it is difficult to interpret these results. Combining such kinetic data with provider kinematic data would provide a more comprehensive overview of ergonomics during the application of MAN and MOB, which could be advantageous for injury prevention.

### Recommendations for reporting provider kinematics during MAN and MOB

In an effort to improve the quality of reporting of provider kinematics during MAN and MOB, we recommend that authors consider the following recommendations during both the design and implementation of their future studies and associated publications. Firstly, there should be an adequate description of the applied procedure. As recommended by Groeneweg and colleagues for MAN [34], several components relating to the delivery of the procedure should be clearly reported, including: i) direction of applied force (e.g., posterior to anterior), ii) velocity of the procedure (e.g., high-velocity, low-amplitude), iii) the ‘name’ of the applied procedure (e.g., side-posture lumbar), iv) the region and level to which the procedure is applied (e.g., L3) and the recipient position (e.g., prone). Furthermore, the template for intervention description and replication (TIDieR) checklist published by Hoffmann and colleagues in 2014 provides a useful guide for: i) authors, to more easily structure the reporting of their interventions; ii) reviewers and editors, to assess the descriptions; and iii) readers, to determine the relevance of the reported results [35]. The combined use of these guidelines would result in such detailed information regarding the applied procedure that it could be accurately replicated in studies conducted by other research groups, thus facilitating a pooling of data and subsequent statistical analysis in the future. Such an analysis was not possible in the current study due to the heterogeneity of the reported data.

Secondly, the individual applying the procedure should also be clearly described. Such information should include their body morphology (e.g., height and weight), training (e.g., physiotherapist, chiropractor) and experience delivering the procedure (e.g., > 5 years for clinicians and ‘X’ hours of classroom experience delivering the procedure). Thirdly, as there is considerable variability in the reported provider kinematics during the delivery of MAN and MOB, it is suggested that authors publish and/or make available raw data (i.e., non-analysed/non-averaged) to support their results where possible and that ranges are reported alongside other descriptive statistics (e.g., mean and standard deviation) for all reported variables, allowing for a more illustrative description of the delivered procedure and investigated variables.

Additionally, recipient body morphology should also be clearly reported. Finally, a detailed description of all measurement equipment used, marker placement, data processing details and the relevant accompanying metrological information (e.g., calibration protocols and outcomes) should also be published in an attempt to facilitate the comparison of data across multiple studies. To ensure that editorial requirements (e.g., word limits) are fulfilled, all these data could feasibly be reported in appendices/supplementary files.

### Limitations

Limitations of the current study include that only manuscripts published in English were included in the search strategy. Additionally, as this study was conducted as a scoping, rather than systematic, review it is possible that some manuscripts reporting on provider kinematics during MAN and/or MOB were not captured by the search strategy. However, we attempted to avoid this situation by employing a broad search strategy inclusive of several professions that routinely use MAN and MOB, conducted across numerous relevant databases, consulting with experienced health sciences librarians, piloting and refinement of the search strategy prior to implementation, and the scoping review was conduced in a systematic fashion (i.e., using two independent reviewers and data extractors). As such, it is unlikely that any seminal study was missed. Furthermore, this review reports only on provider kinematics during MAN and MOB and does not report on the kinematics of the recipient (e.g., movements induced in the participant by the procedure) nor on the procedure kinetics (e.g., force application). Finally, the final stage of the Arksey and O’Malley scoping review framework (optional consultation process) [22] was not included as it was deemed to be unnecessary in the context of the current study.

## Conclusion

There are a small number of studies reporting heterogeneously on the kinematics of providers during the delivery of MAN and MOB. Clear reporting of the body morphology of both the provider and recipient, the applied procedure from a biomechanical perspective (e.g., direction of force application) and of the measurement equipment used could enable future meta-analysis of provider kinematic data during the delivery of MAN and MOB. Such detailed reporting would also facilitate the use of data reporting on provider kinematics in the development of MAN and MOB technique skills curricula and could feasibly be used to mitigate risk for providers.

## Electronic supplementary material

Below is the link to the electronic supplementary material.

Supplementary Material 1

Supplementary Material 2

## Data Availability

The datasets used and/or analysed during the current study are available from the corresponding author on reasonable request.

## Abbreviations

ARV: axial rotation velocity
CINAHL: Cumulative Index to Nursing and Allied Health Literature database
GRF: ground reaction forces
ICL: Index to Chiropractic Literature database
IMU: inertial motion unit
MAN: spinal manipulation
MEDLINE: Medical Literature Analysis and Retrieval System Online database
MeSH: Medical Subject Heading
MLV: maximum lateral velocity
mm: millimeters
MOB: spinal mobilization
MoCap: motion capture
MSF: maximum sagittal flexion
N: Newtons
n: number of studies
PEDro: Physiotherapy Evidence database
PRISMA-ScR: Preferred Reporting Items for Scoping Reviews statement
SD: standard deviation
TIDier: Template for Intervention Description and Replication
