# Supplementary material for: Provider kinematic strategies during the delivery of spinal manipulation and mobilization: a scoping review of the literature
Source: Chiropr Man Therap. 2025 Jan 6;33:1. doi: 10.1186/s12998-024-00564-x (PMC11702080; doi:10.1186/s12998-024-00564-x)
Supplement: Supplementary file 1 — Supplementary Material 1 [file 12998_2024_564_MOESM1_ESM.docx]

# Appendix 1

## MEDLINE search strategy.

| **#** | **Searches** | **Results** |
| --- | --- | --- |
| 1 | Manipulation, Chiropractic/ | 1110 |
| 2 | Manipulation, Spinal/ | 1814 |
| 3 | Manipulation, Orthopedic/ | 3988 |
| 4 | Musculoskeletal Manipulations/ | 2238 |
| 5 | Manipulation, Osteopathic/ | 1213 |
| 6 | (manip* adj3 (chiropr* or osteopath* or naprapath* or spine* or spinal* or manual* or physiotherap* or (physical adj2 therap*) or joint* or lumbar* or lumbo* or back or neck* or cervic* or thorac* or pelvi* or sacr* or orthop* or musculoskeletal* or vertebral* or occip* or HVLA* or low-amplit* or high-velocit* or (high* adj2 veloc*) or (low* adj2 amplit*))).mp. | 13837 |
| 7 | (mobiliz* adj3 (chiropr* or osteopath* or naprapath* or spine* or spinal* or manual* or physiotherap* or (physical adj2 therap*) or joint* or lumbar* or back or neck* or cervic* or thorac* or pelvi* or sacr* or orthop* or therap* or musculoskeletal* or vertebral* or occip* or LVLA or (low adj2 velocit*) or (low* adj2 amplit*) or variable*)).mp. | 2581 |
| 8 | (mobilis* adj3 (chiropr* or osteopath* or naprapath* or spine* or spinal* or manual* or physiotherap* or (physical adj2 therap*) or joint* or lumbar* or back or neck* or cervic* or thorac* or pelvi* or sacr* or orthop* or therap* or musculoskeletal* or vertebral* or occip* or LVLA or (low adj2 velocit*) or (low* adj2 amplit*) or variable*)).ti,ab,kw. | 454 |
| 9 | ((manual therapy or omt or chiropr* or osteopath*) adj3 (skill* or techniqu*)).mp. | 695 |
| 10 | 1 or 2 or 3 or 4 or 5 or 6 or 7 or 8 or 9  **[ ** MANUAL THERAPY/ SPINAL MANIPULATION]** | 16434 |
| 11 | (forc* or biomechanic* or thrust* or kinematic*).ti,ab,kw. | 617361 |
| 12 | ((motor or psychomotor* or practical or palpatory or tactile) adj3 (skill* or techniqu*)).mp. | 43922 |
| 13 | Biomechanical Phenomena/ | 130961 |
| 14 | Physical Phenomena/ | 6103 |
| 15 | Motor Skills/ | 26453 |
| 16 | 11 or 13 or 14 or 15 | 700160 |
| 17 | (manip* adj2 thrust*).ti,ab,kw. | 260 |
| 18 | (forc* or biomechanic* or kinematic*).ti,ab,kw. | 612655 |
| 19 | 17 and 18 **[ *** FORCE/ BIOMECHANICS]** | 62 |
| 20 | 10 and 16 | 1996 |
| 21 | 19 or 20 | 2003 |
| 22 | limit 21 to english language | 1909 |
| 23 | exp Animals/ | 26561407 |
| 24 | exp Humans/ | 21414963 |
| 25 | 23 not 24 | 5146444 |
| 26 | 22 not 25 | 1806 |

## PsychINFO search strategy.

| **#** | **Searches** |
| --- | --- |
| 1 | (manip* adj3 (chiropr* or osteopath* or naprapath* or spine* or spinal* or manual* or physiotherap* or (physical adj2 therap*) or joint* or lumbar* or lumbo* or back or neck* or cervic* or thorac* or pelvi* or sacr* or orthop* or musculoskeletal* or vertebral* or occip* or HVLA* or low-amplit* or high-velocit* or (high* adj2 veloc*) or (low* adj2 amplit*))).mp. |
| 2 | (mobiliz* adj3 (chiropr* or osteopath* or naprapath* or spine* or spinal* or manual* or physiotherap* or (physical adj2 therap*) or joint* or lumbar* or back or neck* or cervic* or thorac* or pelvi* or sacr* or orthop* or therap* or musculoskeletal* or vertebral* or occip* or LVLA or (low adj2 velocit*) or (low* adj2 amplit*))).mp. |
| 3 | (mobilis* adj3 (chiropr* or osteopath* or naprapath* or spine* or spinal* or manual* or physiotherap* or (physical adj2 therap*) or joint* or lumbar* or back or neck* or cervic* or thorac* or pelvi* or sacr* or orthop* or therap* or musculoskeletal* or vertebral* or occip* or LVLA or (low adj2 velocit*) or (low* adj2 amplit*))).mp. |
| 4 | ((manual therapy or omt or chiropr* or osteopath*) adj3 (skill* or techniqu*)).mp. |
| 5 | 1 or 2 or 3 or 4 |
| 6 | biomechanics/ |
| 7 | motor skills/ |
| 8 | Motor Coordination/ |
| 9 | (forc* or biomechanic* or thrust* or kinematic*).ti,ab. |
| 10 | ((motor or psychomotor* or practical or palpatory or tactile) adj3 (skill* or techniqu*)).mp. |
| 11 | 6 or 7 or 8 or 9 or 10 |
| 12 | 5 and 11 |
| 13 | (manip* adj2 thrust*).ti,ab. |
| 14 | (forc* or biomechanic* or kinematic*).ti,ab. |
| 15 | 13 and 14 |
| 16 | 12 or 15 |
| 17 | limit 16 to english language |

## Scopus search strategy.

| **#** | **Searches** |
| --- | --- |
| 1 | ((TITLE(force or forces or kinematic* or biomechanic*)) OR (ABS(force or forces or kinematic* or biomechanic*))) AND ((ABS(( ( manip* OR mobilis* OR mobiliz* ) W/3 ( chiropr* OR osteopath* OR naprapath* OR spine* OR spinal* OR manual* OR physiotherap* OR ( physical W/2 therap* ) OR joint* OR lumbar* OR lumbo* OR back OR neck* OR cervic* OR thorac* OR pelvi* OR sacr* OR orthop* OR musculoskeletal* OR vertebral* OR occip* OR hvla* OR low-amplit* OR high-velocit* OR ( high* W/2 veloc* ) OR ( low* W/2 amplit* ) ) ))) OR (TITLE(( ( manip* OR mobilis* OR mobiliz* ) W/3 ( chiropr* OR osteopath* OR naprapath* OR spine* OR spinal* OR manual* OR physiotherap* OR ( physical W/2 therap* ) OR joint* OR lumbar* OR lumbo* OR back OR neck* OR cervic* OR thorac* OR pelvi* OR sacr* OR orthop* OR musculoskeletal* OR vertebral* OR occip* OR hvla* OR low-amplit* OR high-velocit* OR ( high* W/2 veloc* ) OR ( low* W/2 amplit* ) ) )))) AND ( LIMIT-TO ( SUBJAREA,"MEDI" ) OR LIMIT-TO ( SUBJAREA,"HEAL" ) ) AND ( LIMIT-TO ( DOCTYPE,"ar" ) OR LIMIT-TO ( DOCTYPE,"re" ) ) AND ( LIMIT-TO ( SRCTYPE,"j" ) ) AND ( LIMIT-TO ( LANGUAGE,"English" ) ) AND ( EXCLUDE ( EXACTKEYWORD,"Nonhuman" ) ) |

## Cochrane Library search strategy.

| **#** | **Searches** |
| --- | --- |
| 1 | Manipulation, Chiropractic/ |
| 2 | Manipulation, Spinal/ |
| 3 | Manipulation, Orthopedic/ |
| 4 | Musculoskeletal Manipulations/ |
| 5 | Manipulation, Osteopathic/ |
| 6 | (manip* adj3 (chiropr* or osteopath* or naprapath* or spine* or spinal* or manual* or physiotherap* or (physical adj2 therap*) or joint* or lumbar* or lumbo* or back or neck* or cervic* or thorac* or pelvi* or sacr* or orthop* or musculoskeletal* or vertebral* or occip* or HVLA* or low-amplit* or high-velocit* or (high* adj2 veloc*) or (low* adj2 amplit*))).mp. |
| 7 | (mobiliz* adj3 (chiropr* or osteopath* or naprapath* or spine* or spinal* or manual* or physiotherap* or (physical adj2 therap*) or joint* or lumbar* or back or neck* or cervic* or thorac* or pelvi* or sacr* or orthop* or therap* or musculoskeletal* or vertebral* or occip* or LVLA or (low adj2 velocit*) or (low* adj2 amplit*))).mp. |
| 8 | (mobilis* adj3 (chiropr* or osteopath* or naprapath* or spine* or spinal* or manual* or physiotherap* or (physical adj2 therap*) or joint* or lumbar* or back or neck* or cervic* or thorac* or pelvi* or sacr* or orthop* or therap* or musculoskeletal* or vertebral* or occip* or LVLA or (low adj2 velocit*) or (low* adj2 amplit*))).mp. |
| 9 | Biomechanical Phenomena/ |
| 10 | Physical Phenomena/ |
| 11 | Motor Skills/ |
| 12 | (manip* adj2 thrust*).ti,ab,kw. |
| 13 | (forc* or biomechanic* or kinematic*).ti,ab,kw. |
| 14 | 12 and 13 |
| 15 | (force or forces or biomechanic* or kinematic*).ti,ab. |
| 16 | ((motor or psychomotor* or practical or palpatory or tactile) adj3 (skill* or techniqu*)).ti,ab. |
| 17 | 1 or 2 or 3 or 4 or 5 or 6 or 7 or 8 |
| 18 | (task adj2 force).mp. [mp=title, original title, abstract, floating sub-heading word, mesh headings, heading words, keyword] |
| 19 | (comment or clinical conference or congress or consensus development conference or conference proceeding or editorial or letter).pt. |
| 20 | 9 or 10 or 11 or 15 or 16 |
| 21 | 17 and 20 |
| 22 | 14 or 21 |
| 23 | 22 not 18 |
| 24 | 23 not 19 |
| 25 | limit 24 to medline records |
| 26 | 24 not 25 |

## EMBASE search strategy.

| **#** | **Searches** |
| --- | --- |
| 1 | chiropractic manipulation/ |
| 2 | spine manipulation/ |
| 3 | orthopedic manipulation/ |
| 4 | musculoskeletal manipulation/ |
| 5 | osteopathic manipulation/ |
| 6 | (manip* adj3 (chiropr* or osteopath* or naprapath* or spine* or spinal* or manual* or physiotherap* or (physical adj2 therap*) or joint* or lumbar* or lumbo* or back or neck* or cervic* or thorac* or pelvi* or sacr* or orthop* or musculoskeletal* or vertebral* or occip* or HVLA* or low-amplit* or high-velocit* or (high* adj2 veloc*) or (low* adj2 amplit*))).mp. |
| 7 | (mobiliz* adj3 (chiropr* or osteopath* or naprapath* or spine* or spinal* or manual* or physiotherap* or (physical adj2 therap*) or joint* or lumbar* or back or neck* or cervic* or thorac* or pelvi* or sacr* or orthop* or therap* or musculoskeletal* or vertebral* or occip* or LVLA or (low adj2 velocit*) or (low* adj2 amplit*))).mp. |
| 8 | (mobilis* adj3 (chiropr* or osteopath* or naprapath* or spine* or spinal* or manual* or physiotherap* or (physical adj2 therap*) or joint* or lumbar* or back or neck* or cervic* or thorac* or pelvi* or sacr* or orthop* or therap* or musculoskeletal* or vertebral* or occip* or LVLA or (low adj2 velocit*) or (low* adj2 amplit*))).mp. |
| 9 | ((manual therapy or omt or chiropr* or osteopath*) adj3 (skill* or techniqu*)).mp. |
| 10 | 1 or 2 or 3 or 4 or 5 or 6 or 7 or 8 or 9 |
| 11 | biomechanics/ |
| 12 | mechanics/ |
| 13 | force/ |
| 14 | kinematics/ |
| 15 | physical phenomena/ |
| 16 | motor performance/ |
| 17 | (forc* or biomechanic* or thrust* or kinematic*).ti,ab,kw. |
| 18 | ((motor or psychomotor* or practical or palpatory or tactile) adj3 (skill* or techniqu*)).ti,ab,kw. |
| 19 | 11 or 12 or 13 or 14 or 15 or 16 or 17 or 18 |
| 20 | 10 and 19 |
| 21 | (manip* adj2 thrust*).ti,ab,kw. |
| 22 | (forc* or biomechanic* or kinematic*).ti,ab,kw. |
| 23 | 21 and 22 |
| 24 | 20 or 23 |
| 25 | limit 24 to english language |
| 26 | exp animal/ |
| 27 | exp human/ |
| 28 | 26 not 27 |
| 29 | 25 not 28 |
| 30 | (books or chapter or conference abstract or conference paper or conference review or editorial or letter).pt. |
| 31 | 29 not 30 |
| 32 | limit 31 to "remove medline records" |

## Web of Science search strategy.

| **#** | **Searches** |
| --- | --- |
| 1 | AB=(manip* near/3 (chiropr* or osteopath* or naprapath* or spine* or spinal* or manual* or physiotherap* or (physical near/2 therap*) or joint* or lumbar* or lumbo* or back or neck* or cervic* or thorac* or pelvi* or sacr* or orthop* or musculoskeletal* or vertebral* or occip* or HVLA* or low-amplit* or high-velocit* or (high* near/2 veloc*) or (low* near/2 amplit*)).) |
| 2 | AB=(mobiliz* near/3 (chiropr* or osteopath* or naprapath* or spine* or spinal* or manual* or physiotherap* or (physical near/2 therap*) or joint* or lumbar* or lumbo* or back or neck* or cervic* or thorac* or pelvi* or sacr* or orthop* or musculoskeletal* or vertebral* or occip* or HVLA* or low-amplit* or high-velocit* or (high* near/2 veloc*) or (low* near/2 amplit*)).) |
| 3 | AB=(mobilis* near/3 (chiropr* or osteopath* or naprapath* or spine* or spinal* or manual* or physiotherap* or (physical near/2 therap*) or joint* or lumbar* or lumbo* or back or neck* or cervic* or thorac* or pelvi* or sacr* or orthop* or musculoskeletal* or vertebral* or occip* or HVLA* or low-amplit* or high-velocit* or (high* near/2 veloc*) or (low* near/2 amplit*)).) |
| 4 | #3 OR #2 OR #1 |
| 5 | AB=(force or forces or kinematic* or biomechanic*) |
| 6 | #4 AND #5 |
| 7 | #4 AND #5 and Rehabilitation (Research Areas) |

## PEDro search strategy.

| **#** | **Searches** |
| --- | --- |
| 1 | Chiropractic manipulation AND force |
| 2 | Chiropractic manipulation AND biomechanic |
| 3 | Chiropractic manipulation AND kinematic |
| 4 | Spinal manipulation AND force |
| 5 | Spinal manipulation AND biomechanic |
| 6 | Spinal manipulation AND kinematic |
| 7 | Spinal mobilization AND force |
| 8 | Spinal mobilization AND biomechanic |
| 9 | Spinal mobilization AND kinematic |
| 10 | Spinal mobilisation AND force |
| 11 | Spinal mobilisation AND biomechanic |
| 12 | Spinal mobilisation AND kinematic |

## Index to Chiropractic Literature search strategy.

| **#** | **Searches** |
| --- | --- |
| 1 | Subject:\"Manipulation, Cervical\" OR Subject:\"Manipulation, Chiropractic\" OR Subject:\"Manipulation, Joint\" OR Subject:\"Manipulation, Lumbar\" OR Subject:\"Manipulation, Spinal\" OR Subject:\"Musculoskeletal Manipulations\" OR Subject:\"Manipulation, Thoracic\" OR Subject:\"Manipulation, Orthopedic\" OR Subject:\"Manipulation, Osteopathic\" OR Subject:\"Manipulation, Pelvic\" OR All Fields:manipulation OR All Fields:mobilization OR All Fields:mobilisation  AND Subject:\"Biomechanical Phenomena\" OR Subject:\"Physical Phenomena\" OR Subject:\"Motor Skills\" OR All Fields:force OR All Fields:forces OR All Fields:biomechanics OR All Fields:biomechanical OR All Fields:biomechanic OR All Fields:kinematic OR All Fields:kinematics |

## CINAHL search strategy.

| **#** | **Searches** |
| --- | --- |
| S1 | (MH "Manipulation, Chiropractic") |
| S2 | (MH "Manipulation, Orthopedic") |
| S3 | (MH "Manipulation, Orthopedic") |
| S4 | (MH "Manipulation, Osteopathic") |
| S5 | (MH "Motor Skills") |
| S6 | (MH "Task Performance and Analysis") |
| S7 | (forc* or biomechanic* or thrust* or kinematic*) |
| S8 | (manip* n3 (chiropr* or osteopath* or naprapath* or spine* or spinal* or manual* or physiotherap* or (physical n2 therap*) or joint* or lumbar* or lumbo* or back or neck* or cervic* or thorac* or pelvi* or sacr* or orthop* or musculoskeletal* or vertebral* or occip* or HVLA* or low-amplit* or high-velocit* or (high* n2 veloc*) or (low* n2 amplit*))) |
| S9 | (mobiliz* n3 (chiropr* or osteopath* or naprapath* or spine* or spinal* or manual* or physiotherap* or (physical n2 therap*) or joint* or lumbar* or back or neck* or cervic* or thorac* or pelvi* or sacr* or orthop* or therap* or musculoskeletal* or vertebral* or occip* or LVLA or (low n2 velocit*) or (low* nj2 amplit*) or variable*)) |
| S10 | (mobilis* n3 (chiropr* or osteopath* or naprapath* or spine* or spinal* or manual* or physiotherap* or (physical n2 therap*) or joint* or lumbar* or back or neck* or cervic* or thorac* or pelvi* or sacr* or orthop* or therap* or musculoskeletal* or vertebral* or occip* or LVLA or (low n2 velocit*) or (low* nj2 amplit*) or variable*)) |
| S11 | ((manual therapy or omt or chiropr* or osteopath*) n3 (skill* or techniqu*)) |
| S12 | S1 OR S2 OR S3 OR S4 OR S8 OR S9 OR S10 OR S11 |
| S13 | ((motor or psychomotor* or practical or palpatory or tactile) n3 (skill* or techniqu*)) |
| S14 | S5 OR S6 OR S7 OR S13 |
| S15 | S12 AND S14 |
| S16 | (manip* n2 thrust*) |
| S17 | (forc* or biomechanic* or kinematic*) |
| S18 | S16 AND S17 |
| S19 | S15 OR S18 |
| S20 | PT (abstract or brief item or book review or commentary or doctoral dissertation or editorial or letter or proceedings) |
| S21 | s19 NOT s20 |
|  | Limiters - English Language; Exclude MEDLINE records |
