# Supplementary material for: Provider kinematic strategies during the delivery of spinal manipulation and mobilization: a scoping review of the literature
Source: Chiropr Man Therap. 2025 Jan 6;33:1. doi: 10.1186/s12998-024-00564-x (PMC11702080; doi:10.1186/s12998-024-00564-x)
Supplement: Supplementary file 2 — Supplementary Material 2 [file 12998_2024_564_MOESM2_ESM.docx]

# Appendix 2

## Articles that were excluded at full-text review

1. Adams AA, Wood J. Comparison of forces used in selected adjustments of the low back by experienced chiropractors and chiropractic students with no clinical experience: a preliminary study. Res Forum. 1984 Autumn;1(1):16–23.
   - Ineligible outcome
2. Adams AA, Wood J. Changes in force parameters with practice experience for selected low back adjustments. Res Forum. 1985 Winter;1(2):40–8.
   - Ineligible outcome
3. Ammer K. Kinematics of two cervical spine manipulative procedures. Manuelle Medizin. 2013;51(3):244–6.
   - Ineligible study design
   - Ineligible language
4. Atkinson BW, Maher T. Thumb Pain in Physiotherapists: Biomechanical Causes of Pain and Alternate Methods of Preventing Distress in Treatment. Journal of Manual & Manipulative Therapy. 2004 Oct 1;12(4):187–91.
   - Ineligible study design
5. Brown L. An Introduction to the Treatment and Examination of the Spine by Combined Movements. Physiotherapy. 1988 Jul 10;74(7):347–53.
   - Ineligible study design
6. Buckingham G, Das R, Trott P. Position of undergraduate students’ thumbs during mobilisation is poor: an observational study. Aust J Physiother. 2007;53(1):55–9.
   - Ineligible outcome
7. Cuesta-Vargas AI, Williams J. Inertial sensor real-time feedback enhances the learning of cervical spine manipulation: a prospective study. BMC Med Educ. 2014 Jun 19;14:120.
   - Ineligible outcome
8. Dugailly PM, Sobczak S, Van Geyt B, Bonnechère B, Maroye L, Moiseev F, et al. Head-Trunk Kinematics During High-Velocity–Low-Amplitude Manipulation of the Cervical Spine in Asymptomatic Subjects: Helical Axis Computation and Anatomic Motion Modeling. Journal of Manipulative & Physiological Therapeutics. 2015;38(6):416–24.
   - Ineligible outcome
9. Dugailly PM, Michaud A, Feipel V, Beyer B. Reaction force magnitude and orientation during supine thoracic spine thrust manipulation: an exploratory analysis and reliability of preload and impulse phase. J Manipulative Physiol Ther. 2020 Jul;43(6):597–605.
   - Ineligible outcome
10. Engell S, Triano JJ, Howarth SJ. Force transmission between thoracic and cervical segments of the spine during prone-lying high-velocity low-amplitude spinal manipulation: A proof of principle for the concept of regional interdependence. Clin Biomech. 10;69:58–63.
    - Ineligible outcome
11. Gelley GM, Passmore SR, MacNeil BJ. Acceleration of clinician hand movements during spinal manipulative therapy. Manual Ther. 2015 Apr;20(2):342–8.
    - Ineligible outcome
12. Graham BA, Clausen P, Bolton PS. A descriptive study of the force and displacement profiles of the toggle-recoil spinal manipulative procedure (adjustment) as performed by chiropractors. Manual Ther. 2010 Feb;15(1):74–9.
    - Ineligible outcome
13. Gudavalli MR. Instantaneous rate of loading during manual high-velocity, low-amplitude spinal manipulations. J Manipulative Physiol Ther. 2014 Jun;37(5):294–9.
    - Ineligible outcome
14. Hammond FL, Talbot SG, Wood RJ, Howe RD. Measurement System for the Characterization of Micro-Manipulation Motion and Force. Journal of Medical Devices. 2013;7(030940).
    - MAN/MOB not applied
    - Ineligible study design
15. Hu MT, Hsu AT, Su FC. Kinematic Analyses of the Thumb during Simulated Posteroanterior Glide Mobilization. PLoS ONE. 2016;11(9):e0161624.

- MAN/MOB not applied

1. Huang X, Lin D, Liang Z, Deng Y, He Z, Wang M, et al. Mechanical Parameters and Trajectory of Two Chinese Cervical Manipulations Compared by a Motion Capture System. Frontiers in Bioengineering and Biotechnology. 2021;9.
   - Ineligible outcome
2. Huijbregts P, Young S. Adding spinal thrust manipulation to entry-level Canadian physical therapy curricula: why and how? Physiotherapy Canada. 2007 Winter;59(1):1–4.
   - Ineligible study design
3. Joo S, Kim J, Lee Y, Song C. The biomechanical analysis of magnitude and direction of force by different techniques of thoracic spinal manipulation. Biomed Res Int. 2020;2020:8928071.
   - Ineligible outcome
4. Lee TH, Cheng TS. Effects of hand posture, breathing type, arm posture and body posture on hand errors. Int J Occup Saf Ergon. 2012;18(3):393–8.

- MAN/MOB not applied

1. Liguo Z, Minshan F, Xunlu Y, Shangquan W, Jie Y. Kinematics Analysis of Cervical Rotation-Traction Manipulation Measured by a Motion Capture System. Evidence-based Complementary and Alternative Medicine. 2017;2017.
   - Ineligible outcome
2. Marchand AA, Mendoza L, Dugas C, Descarreaux M, Page I. Effects of practice variability on spinal manipulation learning. J Chiropractic Educ. 2017 Oct;31(2):90–5.
   - Ineligible outcome
3. Öten E, Uğur L. Investigation of postero-anterior mobilization in the lumbar spine: A finite element analysis study. Journal of Surgery & Medicine. 2022;6(3):356–9.

- MAN/MOB not applied
- Ineligible study design

1. Tsuji S, Tsujimura H, Shirahoshi SI, Taoda K, Kitahara T. Effects of different bed heights on the physical burden of physiotherapists during manual therapy: an experimental study. Ind Health. 2023 Jun 3;61(3):213–21.

- MAN/MOB not applied
- Ineligible outcome

1. Van Geyt B, Dugailly PMA, De Page L, Feipel V. Relationship Between Subjective Experience of Individuals, Practitioner Seniority, Cavitation Occurrence, and 3-Dimensional Kinematics During Cervical Spine Manipulation. Journal of Manipulative and Physiological Therapeutics. 2017;40(9):643–8.
   - Ineligible outcome
2. Viner A, Lee M. Direction of manual force applied during assessment of stiffness in the lumbosacral spine. J Manipulative Physiol Ther. 1995 Sep;18(7):441–7.
   - Ineligible outcome
3. Walsh T, Delahunt E, McCarthy Persson U. Effects of taping on thumb alignment and force application during PA mobilisations. Man Ther. 2011;16(3):264–9.

- MAN/MOB not applied

1. Watson TA, Radwan H. Comparison of three teaching methods for learning spinal manipulation skill: a pilot study. Journal of manual & manipulative therapy. 2001;9(1):48‐52.
   - Ineligible outcome
2. Williams JM, Cuesta-Vargas A. Quantification of prone thoracic manipulation using inertial sensor-derived accelerations. J Manipulative Physiol Ther. 2014 May;37(4):230–5.
   - Ineligible outcome
3. Zhang Q, Xie Q, Liu H, Sheng B, Xiong S, Zhang Y. A pilot study of biomechanical and ergonomic analyses of risky manual tasks in physical therapy. International Journal of Industrial Ergonomics. 2022;89.
   - Ineligible outcome
